# Surface Quality as a Factor Affecting the Functionality of Products Manufactured with Metal and 3D Printing Technologies

**DOI:** 10.3390/ma17215371

**Published:** 2024-11-02

**Authors:** Maria Richert, Marek Dudek, Dariusz Sala

**Affiliations:** Management Faculty, AGH University, Gramatyka 10 str., 30-067 Kraków, Poland; madudek@agh.edu.pl (M.D.); sala@agh.edu.pl (D.S.)

**Keywords:** surface engineering, functional coatings, nanomaterials, 3D printing, metals

## Abstract

Surface engineering is one of the most extensive industries. Virtually all areas of the economy benefit from the achievements of surface engineering. Surface quality affects the quality of finished products as well as the quality of manufactured parts. It affects both functional qualities and esthetics. Surface quality affects the image and reputation of a brand. This is particularly true for cars and household appliances. Surface modification of products is also aimed at improving their functional and protective properties. This applies to surfaces for producing hydrophobic surfaces, anti-wear protection of friction pairs, corrosion protection, and others. Metal technologies and 3D printing benefit from surface technologies that improve their functionality and facilitate the operation of products. Surface engineering offers a range of different coating and layering methods from varnishing and painting to sophisticated nanometric coatings. This paper presents an overview of selected surface engineering issues pertaining to metal products, with a particular focus on surface modification of products manufactured by 3D printing technology. It evaluates the impact of the surface quality of products on their functional and performance qualities.

## 1. Introduction

Products must have the right characteristics to meet the established quality standards. One of the important parameters affecting the quality of products is the quality of their surface. This parameter often determines the demand for the product [1,2]. Much attention with regard to surface quality is paid to metal products [3,4,5]. This applies to numerous products and pieces of equipment commonly used in industries, as well as in households.

Innovative methods of refining metal surfaces make it possible to achieve new properties [6]. These methods are related to the fabrication of nanostructures, coating of surfaces with coatings produced by thermal spray methods, and the production of coatings by chemical vapor deposition (CVD) or physical vapor deposition (PVD). Chemical or physical surface treatment is also used. Among them are cutting, grinding, ion implantation, laser surface treatment, spraying, electrolytic polishing, painting, and others. An overview study by Ramezani et al. [7] presents state-of-the-art coating manufacturing methods and the possibilities for assessing their quality.

Applying layers and coatings involves appropriate preparation of the surface of the substrate on which the coating is to be deposited. The morphology of the substrate surface is of great importance. Thermal spray methods require appropriate surface roughness due to the mechanical bonding of the coating particles with the substrate. In turn, in PVD and CVD methods the substrates should be not only clean but also smooth because the coating reflects the surface unevenness. Metals become oxidized and contaminated during product manufacturing processes. Before depositing coatings, it is necessary to clean the surface of oils, fats, and corrosion products. Various cleaning methods are used, usually adapted to the coating deposition technology. These may include chemical cleaning processes such as etching, physical cleaning processes such as grinding and polishing, or modern ion beam surface etching processes. The material interphase between the substrate and the deposited coatings is the area that determines the quality of adhesion of the coating to the substrate. Research in this area requires advanced observation techniques such as scanning electron microscopy or transmission electron microscopy. The connection of the coating with the substrate is related to the phenomenon of adhesion. Adhesion only concerns physical surface interactions between particles of matter in contact and is a mechanism that occurs during the deposition of coatings by thermal spray or PVD [8]. Cohesion refers to the attractive forces of particles of the same material and is associated with the growth of coatings and their consistency. A much stronger interaction between molecules is related to the phenomenon of chemisorption, which enables the formation of strong chemical bonds (usually covalent), as opposed to the weak intermolecular Van der Wals interactions occurring in the case of physisorption. Surface phenomena at the interface of phases are important for the mechanisms of coating deposition. Important phenomena determining the formation of the coating, apart from adhesion and chemisorption, include the following: wettability, surface tension, external friction, viscosity, and diffusion.

The ability to modify the surface properties of metals has opened up new possibilities for improving their mechanical strength, wear resistance, corrosion protection, and biocompatibility [9]. Surface engineering methods are being applied in many industrial fields and make it possible to improve the quality of products. Surface engineering applies, among other things, to products manufactured by 3D additive methods, which are one of the underlying production methods in Industry 4.0 and 5.0 [10,11]. Surface quality in products for medicine is also of great importance [12]. Also, surface quality is an important element in the automotive industry [13,14,15,16]. Surface engineering, particularly plasma technology engineering, can play an important role in improving engine performance and reducing vehicle weight [17]. The use of tribological coatings deposited on engine components using plasma technology processes brings many benefits to the automotive industry. This technology can enable the production of vehicles with significantly better performance, greater reliability, and are characterized by more economical operation. Engine parts are coated with coatings such as chromium nitride CrN, Cr_2_N, diamond-like carbon DLC, and nano-laminates like CrN/AlN deposited by PVD methods [18]. The aforementioned types of coatings are also used to coat piston rings, valve lifters, cams, journal bearings, and exhaust valves. Diesel engine cylinder liners are coated with hard chrome for excellent wear resistance and low friction [19].

Significant developments in surface engineering are hard coatings such as nitrides, diamond and diamond-like coatings, and cBN carbon layers. The coatings of the future in engine and powertrain components of automobiles are expected to be thin-film solar cells as self-cleaning and self-healing surfaces [20]. In addition to their favorable tribological properties, diamond-like carbon coatings may also have sensory properties. Modern plasma coating techniques make it possible to produce multifunctional surfaces that are tribologically optimized and yet have sensory capabilities [21]. The aerospace industry is also a significant contributor to the demand for surface enhancement technologies to protect surfaces from erosion, high temperatures, and corrosion [22,23,24]. Some of the latest methods used to coat the surfaces of aerospace components are plasma electrolytic oxidation (PEO), ion implantation, and laser peening [25].

The aerospace industry is one of many that is benefiting greatly from the emergence of 3D printing. Three-dimensional technology makes it possible to produce complex shapes and components that would be difficult to make by conventional means. In addition, it allows the production of rough or permeable surfaces, introducing a new quality to products. The use of 3D technology in aerospace is of great interest and is the subject of research, with a particular focus on the analysis of the state of surfaces after the manufacturing process [26]. Phenomena related to surface cleaning in aviation are presented in the work of Ünaldi et al. [27].

Surface shaping and modeling is a very broad field and includes a wide range of conventional and unconventional methods. Of all aerological systems, almost 50% involve paint coatings [28]. They are very widely used in the automotive industry [29], as well as aerospace [30]. A review paper by Pendar et al. [31] presents the latest developments in automobile surface protection, including data on varnish coating. Currently, the surface of end products is coated with at least five layers to produce a coating with different functions and thicknesses. Each of the five coating stages involves a curing or drying process. Special coatings are used to protect against corrosion and erosion [32]**.** Much of the work focuses on studying the degradation and performance of paint coatings in the automotive industry. The work of Hernández-Peña et al. shows the mechanisms and erosion resistance of automotive paint coatings after laboratory testing of their accelerated aging [33]. A review paper by Akafuah et al. presents a hybrid technology for producing protective coatings on car bodies [34]. The study of bonded joints is one of the issues complementing the application possibilities of surface engineering technology [35].

This article contains six chapters presenting modern coating technologies and their application in the area of metal products, with particular emphasis on 3D technology. The first chapter is an introduction to the issue of product surface quality. The second one describes and characterizes new surface coating technologies and illustrates the structures of the produced coatings. The third chapter presents ways of modifying the surfaces of metal products with 3D technology.

In chapter four, the impact of surface quality on the functional characteristics of metal products manufactured using conventional technologies and 3D printing is explored. The surface engineering market is presented in chapter five. The sixth chapter is a summary of the article.

## 2. Novel Surface Coating Technologies

Various technologies are used to protect and refine surfaces. Some of the most modern methods include PVD [36,37,38], CVD [39,40,41,42,43], and thermal spray coating (TSC) [44]. Galvanic surface coating, which has been used for years, is still useful [45] (Table 1).

Coatings deposited using these methods differ in their internal structural structure, which affects their properties. The structure of coatings deposited using PVD and CVD methods is columnar. The coating material grows successively from the substrate by attaching successive particles made of single atoms produced from appropriate precursors. In turn, thermally sprayed coatings are composed of grains elongated along the surface of the substrate. The grain structure of the coating is created as a result of the impact of a stream of molten particles on the substrate. The TSC coating, in addition to elongated grains, contains unmelted particles, pores, and discontinuities. TSC coatings are characterized by significant porosity, which results in lower hardness compared to PVD and CVD coatings. For example, the hardness of a TiC coating deposited using the CVD method is approximately HV = 3700 [46]. Colombo-Pulgarín et al. provide the hardness of a TiN coating deposited using the CVD method, HV = 1355 [47]. The hardness of coatings deposited using the PVD method is in the range of HV = 1500–4500 [48], while the hardness of thermally sprayed coatings is in the range of HV = 750–1400 [49,50,51].

PVD processing can be divided into four categories: Vacuum Deposition, Sputter Deposition, Arc Vapor Deposition, and Ion Plating. The main varieties of PVD are as follows:

Cathodic Arc Deposition or ARC-PVD is a physical vapor deposition technique in which an electric arc is used to vaporize material from a cathode target. The vaporized material then condenses on a substrate, forming a thin film. The technique can be used to deposit metallic, ceramic, and composite films.

Cathodic ARC Deposition (CAD), also called vacuum arc deposition, has been developed as a competitive ion plating technique.

Vacuum Evaporation is a foundational PVD technique where materials (referred to as the evaporating material) are heated in a vacuum to the point where they evaporate and then condense on a target substrate to form a thin film.

Sputtering Deposition is a highly versatile PVD method that involves ejecting material from a target (or “sputter target“) through bombardment with energetic particles, usually ions, which then deposit onto a substrate to form a thin film. This method can be adapted to deposit a wide range of materials including metals, ceramics, and plastics.

Plasma Spray Coating is a PVD technique that utilizes a high-temperature plasma jet to melt and propel materials onto a substrate, forming a coating. This method is particularly effective for applying thick coatings over large surface areas and is highly versatile in terms of the materials it can process.

Ion Plating is a sophisticated PVD technique that enhances the adhesion and quality of thin films through the use of ionized vapor particles, which are accelerated towards the substrate under an electric field. This method is renowned for producing highly durable and adherent coatings, making it ideal for both functional and decorative applications.

Pulsed Laser Deposition (PLD) is a versatile PVD method that uses high-power laser pulses to vaporize material from a target, which then deposits on a substrate to form a thin film. This method is particularly favored for its ability to deposit a wide range of materials with precise control over the film’s composition and thickness.

Electron Beam Physical Vapor Deposition (EBPVD) is a specialized PVD technique that uses an electron beam to heat and vaporize the target material in a vacuum, resulting in high-quality, pure thin films. This method is particularly effective for materials with high melting points and for applications requiring precise control over film properties.

**Figure 1:**
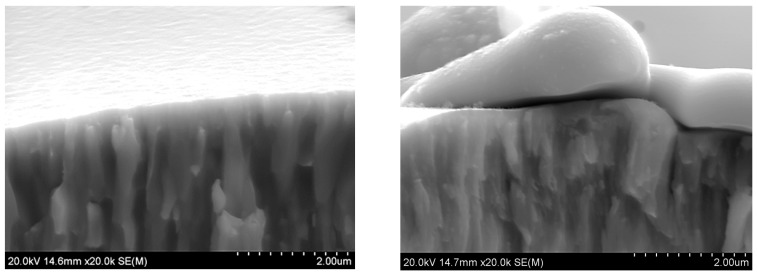
Structure of a molybdenum coating deposited by PVD with a top DLC layer, deposited by CVD method.

Spraying coatings using plasma methods is one of the most modern surface engineering solutions and is currently used on an industrial scale. PVD, which is one of these techniques, is used to cover the nitrided surfaces of forging dies with abrasion-resistant layers [52]. The PVD method is also used to cover nitrided dies for stamping and extruding metals and alloys, as well as other tools and devices exposed to abrasive wear. CVD technology is also widely used to cover surfaces with coatings such as TiN, TiCrN, Al_2_O_3_, and DLCs—diamond-like coatings (Figure 1). The resulting layer is several micrometers wide. The thickness of PVD layers is usually 1–10 mm.

**Figure 2:**
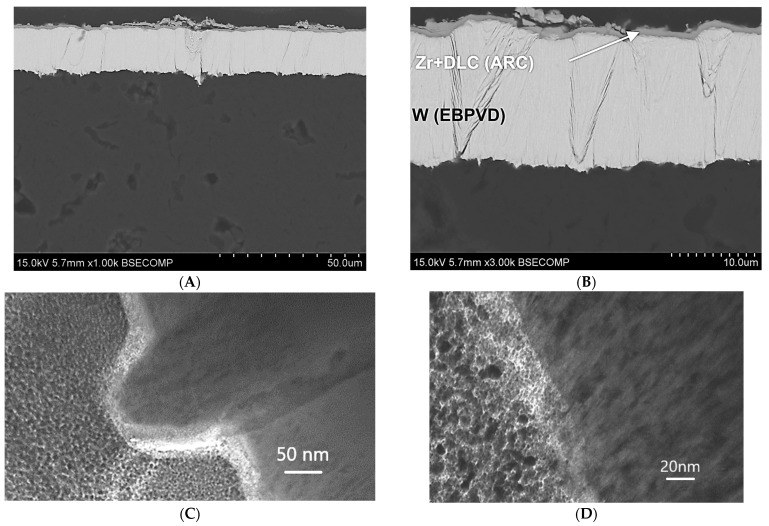
Nanometric structure of tungsten coating (EBPVD) + (Zr + DLC) (Arc PVD). (**A**,**B**) scanning electron microscope observations and (**C**,**D**) transmission electron microscope observations.

The properties of the coating depend on its internal microstructure. In particular, if the coating structure has nanometric dimensions, its hardness and strength increases. Examples of the structures of nanometric coatings are presented in Figure 2, which shows a tungsten coating deposited on a substrate using the EBPVD method and a Zr layer with diamond-like carbon deposited on this coating using the ARC PVD method. The structure of the coatings is columnar, which results from the mechanism of their formation. The coatings grow from the substrate, which in this case was an Al-Si aluminum alloy. During coating deposition, the coating material is evaporated from the target. The evaporated metal ions of the coating are connected to the substrate by the applied voltage. Initially, ion deposition is island-like and, as it progresses, the columns join and a compact structure is formed. Figure 2C,D shows the interphase area between the substrate and the coating. A detailed study of this area would require much higher image resolution and magnifications. However, the visible change in contrast in this area indicates a border transition region connecting the substrate to the coating. The connection requires matching materials with different crystal lattices. Electron microscopic examination of thin films prepared from cross-sections of this coating revealed that the basic elements of the microstructure have nanometric dimensions. The sizes of the particles from which the columnar structures are composed were below 20 nm.

The latest achievements in surface engineering are intelligent coatings [53]. These are innovative coatings that spontaneously respond to external stimuli and thus provide added value beyond basic, passive functions such as decoration or surface protection. The stimuli initiating this type of coating may be heat, light radiation, mechanical induction, temperature, pressure, pH fluctuations, aggressive corrosive ions, and many others.

Therefore, smart coatings are also called “stimulus-responsive” or “environmentally sensitive” coatings [54]. Smart coatings include self-cleaning surfaces that use photocatalytic reactions and surfaces with controlled transparency and emission of heat and light transfer [55].

**Figure 3:**
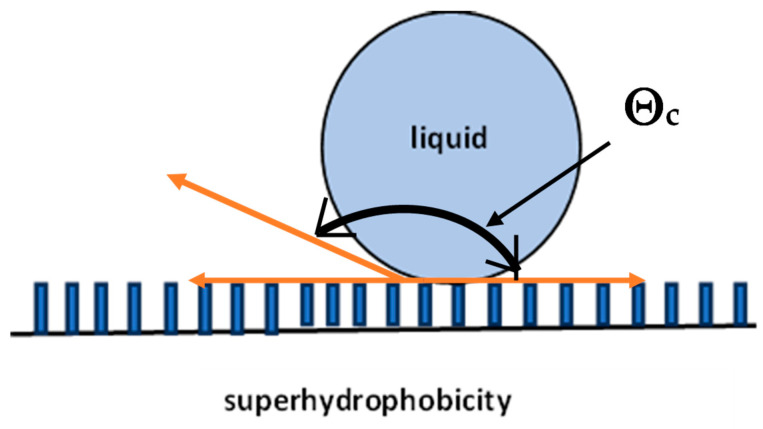
Superhydrophobic surface with contact angle Θ_c_ > 150°.

**Figure 4:**
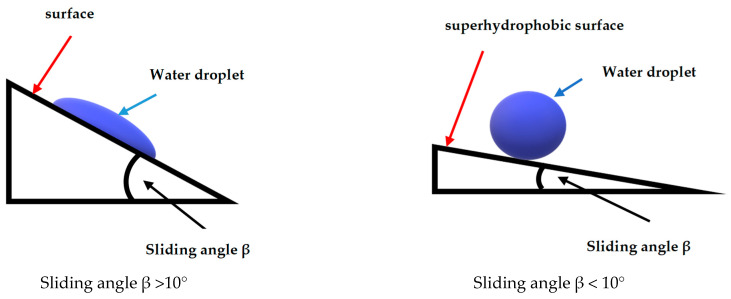
The sliding angle β in different wettability levels.

**Figure 5:**
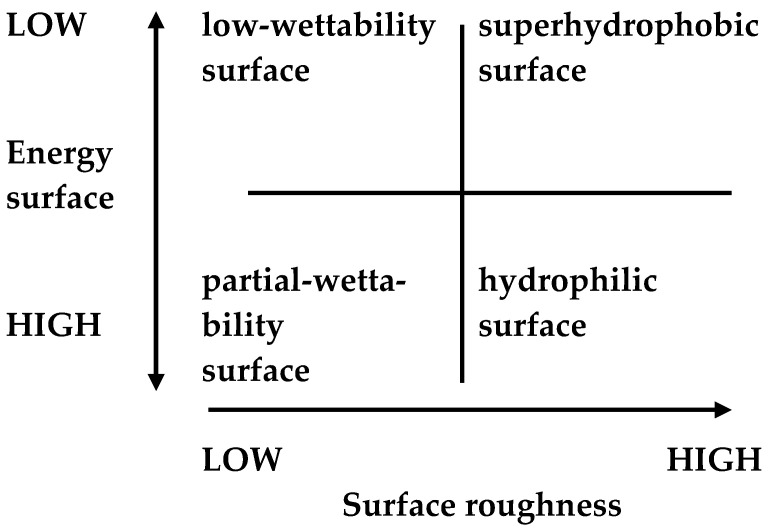
Wettability conditions.

Regarding wettability, the contact angle is formed by the surface and the plane tangent to the liquid surface. The greater the contact angle, the greater the degree of hydrophobicity [56,57]. On a surface with a contact angle of 180°, a drop of water forms a perfect sphere. Generally, hydrophobic surfaces have a contact angle of 90 to 180° (Figure 3). In the case of superhydrophobicity, in addition to the contact angle above 150 degrees, water should flow freely with a small slight tilting angle of several degrees.This slight tilting angle of the surface is known as the sliding angle (Figure 4) [58]. For a superhydrophobic surface, this sliding surface should be very low, generally less than 6 degrees [59]. Surface wettability depends largely on the chemical composition and microscopic surface geometry of the materials. It is influenced by the physical roughness and chemical heterogeneity as well as the surface energy of the materials (Figure 5). For a surface to achieve superhydrophobicity, two main conditions must be fulfilled: a low surface energy and hierarchical structure of the surface on the nano/microscale (high surface roughness on the nanoscale).

Recent research on superhydrophobic and icephobic surfaces is inspired by nature. Lotus leaves have a hydrophobic surface, which has a low surface energy and is formed as a result of a combination of hierarchical microstructures and nanostructures. The fabrication of synthetic superhydrophobic surfaces with lotus leaf-like morphological topography typically involves surface nanostructuring using nanoparticles, photolithography, mesoporous polymers, and surface etching, sometimes combined with chemical modification to reduce the surface energy [60]. Nature-inspired design and fabrication of superhydrophobic coatings involves creating materials with low surface energy and/or rough surface textures (Figure 5). Nanostructural technologies are particularly important when creating superhydrophobic coatings. Coatings based on nanostructures act as intelligent, multifunctional materials. For example, superhydrophobic surfaces with self-cleaning properties are created as a result of the formation of rough structures with a double texture scale, with micro- and nano-roughness (so-called double-hierarchical structures) [61,62,63,64,65].

Smart coatings have the ability to detect and prevent corrosion by influencing corrosion inhibitors [66]. They find wide, diverse applications in industries such as health care, defense, textiles, transportation, construction, electronics, and others. Examples of smart coatings include antibacterial, antifouling, conductive, and superhydrophobic systems. Superhydrophobicity can be fabricated with the help of micro- and nanostructures by different methods such as plasma treatment, the Etching Method, the Sol–Gel Method, the Electrospinning Method, the Electrodeposition Method, chemical vapor deposition, and Chemical Bath Deposition [67]. Controlling the wetting of surfaces is an important problem relevant to many areas of technology. The interest in self-cleaning surfaces is being driven by the desire to fabricate such surfaces for satellite dishes, solar energy panels, photovoltaics, exterior architectural glass and green houses, and heat transfer surfaces in air conditioning equipment [68].

Surface wettability is considered one of the most important parameters influencing the biological response to materials implanted in the body. Wettability influences protein adsorption, platelet adhesion/activation, blood coagulation, and cellular bacterial adhesion [69,70]. In general, cell binding is weak on any hydrophobic surface and high on moderately hydrophilic surfaces. Surface wettability plays an important role in various fields of the economy, medicine, energy, electronics, and construction. Coatings can change the surface wettability and functionality of products. In engineering applications, surface modification by deposition of various types of coatings largely applies to metal surfaces. Products made of steel and non-ferrous metals manufactured using plastic processing methods and, increasingly, new technologies such as 3D printing, obtain better properties after being covered with layers or coatings with special properties enabling their use. Coatings are commonly used on various types of tools, everyday products, instruments, and industrial, transport, and other equipment. Depending on the application, coatings are produced using different methods and have different properties. They are used to protect surfaces against high temperatures, corrosion, or wear or as decorative coatings. Currently, surface engineering is an inherent element of the technological cycle and the final operation of many productions [71]. Not only coatings can refine and change the properties of the outer layers of products. Changes in surface properties can also be achieved by modifying the surface itself. Surface treatments include the following: nitriding, ion implantation, heat treatment, carburizing, surface strengthening by deformation, grinding and polishing, chemical etching, and other materials engineering treatments.

## 3. Surface Modification of Metal Products Manufactured by 3D Printing

Three-dimensional printing is becoming more and more widely used in industry. It allows for rapid prototyping, which shortens the time to introduce new products to the market. Three-dimensional technology is used to produce spare parts, tools, and molds. Three-dimensional printing is being used more and more boldly in the metal industry and creates an alternative to traditional methods of producing metal elements. Currently, this technology enables the production of high-quality elements that can be immediately used in production processes. Metal 3D printing technology enables the production of very complex parts and non-standard elements.

It is believed that so-called surface-functionalized 3D printing, by adding a new functional dimension to the surface of 3D-printed objects, is a cost-effective and highly desirable approach to 3D printing. Such an operation meets a variety of quality requirements for 3D products. Compared to developing special-purpose materials for each individual case, 3D printing definitely saves production time by increasing productivity [72].

Currently, there are developed strategies for surface modification of 3D products by such methods as etching, deposition treatment, micro/nanocomposition coating and surface modification by polymer brush. Surface modification of 3D devices is used in biomedicine, engineering, flexible electronics, smart devices, and other fields due to the characteristics desired in the operation of these devices, such as wetting, biocompatibility and bioactivity, high strength and conductivity, etc.

Of great interest are 3D printing methods for metal parts that allow modification of surface wetting [73]. Selective laser melting (SLM), also defined by ISO/ASTM as Laser Powder Bed Fusion (L-PBF), is widely used to manufacture metal parts. The surface of parts fabricated in this way is rough due to the uneven distribution of laser radiation during fabrication. Manufactured parts have hydrophilic surfaces because metal alloys inherently have high surface energy levels. 

**Figure 6:**
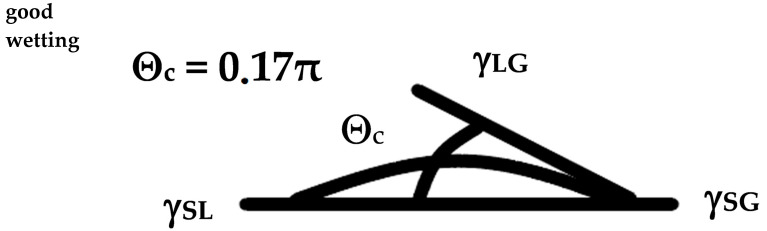

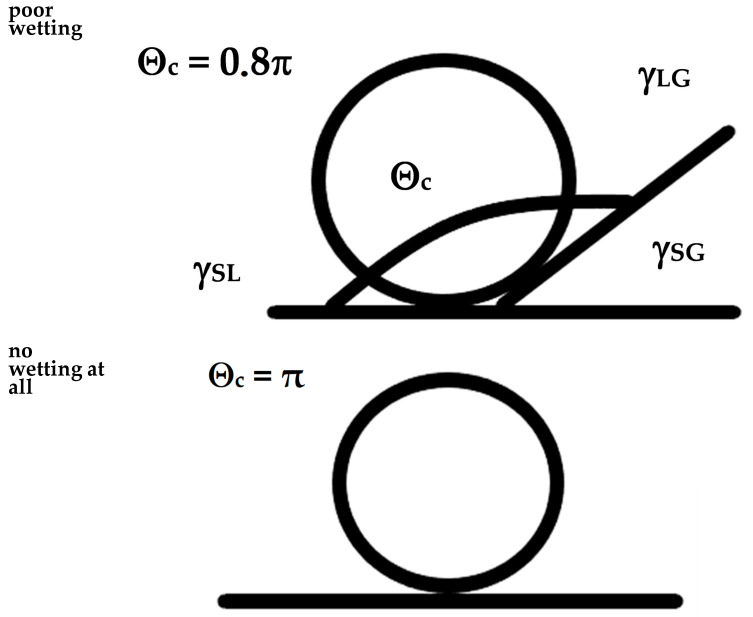
Superhydrophobic surfaces characterized by a contact angle Θc > 150°, γ_SL_—surface tension at the liquid–solid interface; γ_SG—_surface tension at the gas–solid interface; γ_LG—_surface tension at the gas–liquid interface.

The aspiration of research work is to produce superhydrophobic surfaces characterized by a contact angle (CA) Θc > 150° and a sliding angle (SA-β) < 10°, which have a number of practical applications (Figure 6). The wetting of printed parts is usually unstable and can change after surface fabrication and cleaning. Research on stabilizing the wetting has advanced and is concerned with subsequent surface treatment after the part is printed. This involves treating the surface with a specific morphology using chemical reagents.

The state of the system (Figure 6) is described by Young’s equation

γ_SG_ = γ_SL_ + γ_LG cos_Θ_c_

Young’s equation describes the ideal state, as it assumes that the contact surfaces are homogeneous. In the case of rough surfaces, the measured values are subject to errors because the relationship does not take into account the surface structure.

A superhydrophobic surface can also be formed by other methods, avoiding the use of environmentally harmful chemical reagents. This can be achieved by producing nanostructured amorphous carbon by decomposing the polymer PVP [74]. Attempts have also been made to produce hydrophobic surfaces by forming cylindrical micro/nanostructures using μ-SLA (micro-Stereolithography Appearance) [75] additive manufacturing technology and a good hydrophobic effect with the maximum contact angle of 143.6° was obtained [76]. In the work of [77], a thermal shape memory superhydrophobic surface with micro/nanostructures was prepared by 3D FDM PLA printing. To obtain the superhydrophobic layer, a mixture of xylene, SiO_2_, and PDMS was sprayed onto the microplate structures. Superhydrophobic surfaces are expected to have a wide range of applications. Superhydrophobic surfaces are extremely resistant to water. Droplets simply slide off them, which offers great potential in terms of protecting surfaces from moisture and dirt, preventing fogging and icing, and fighting bacteria and other germs. However, superhydrophobic coatings are mechanically fragile. They exhibit low durability and strength. Therefore, further work is needed to improve them mechanically.

Three-dimensional-printed bone implants are constructed of scaffolds that exhibit rather poor bioactivity. Techniques have been developed to modify their surfaces to enhance the poor bioactivity of 3D-printed scaffolds [78]. Several strategies have been proposed, including roughening the surface to increase cell proliferation [79], peptide modification of the scaffold surface [80], surface modification with hyaluronic acid and collagen [81], and the use of clam adhesive proteins for better cell adhesion [82].

Bioengineering is a field in which the use of 3D printing is growing. Among others, dentistry is a significant customer of products produced by 3D printing technology [83]. Laser micromachining is being used to create precise nano- and microscale objects on the surfaces of dental implants. The characteristics of implant surfaces modified by oxidation were studied in a paper by Deppe et al. [84]. It was noted that the choice of surface treatment can affect the properties of implants during the insertion process. The surface of dental implants produced by 3D printing technology was treated using a selective laser melting process, followed by further nanostructuring by electrochemical anodizing to form titanium nanotubes (TNTs) and subsequent bioactivation with the formation of a hydroxyapatite (HA) coating [85]. The results suggest that 3D printing technology combined with electrochemical nanostructuring and HA modification is a promising approach to fabricate Ti implants with improved osteointegration and is a potential alternative to conventional dental implants. The surface quality of 3D-printed Ti implants used in hip joints, among others, is not directly suitable for clinical applications. A novel method for surface modification of 3D-printed Ti-6Al-4V implants is presented by Yu et al. [86]. It involves combining acid etching with hydrothermal treatment to construct micro/nanostructures to improve the osteogenesis ability of Ti implants. Orlowska et al. [87] presented a surface modification of highly porous titanium implants manufactured using additive methods involving plasma electrolytic oxidation. The use of this method improves the corrosion resistance of the implants, reduces the penetration of metal ions into the environment, and creates a better substrate for bone cell adhesion and proliferation.

Recent achievements include superhydrophobic surfaces with self-cleaning and anti-icing properties. Such surfaces are produced on mild steel and are used in aircraft and marine systems [88]. The research results presented in the work of Cheirmakani et al. [89] showed that creating a rough surface on steel (the surface roughness in this study was 4.2 µm) leads to the generation of numerous grooves in which air can be trapped, which increases the contact angle. The surface contact angle obtained by the researchers was 154°. In this way, without applying a coating, a hydrophobic effect was achieved only by mechanical treatment of the surface.

## 4. The Impact of Surface Quality on the Functional Characteristics of Metal Products Manufactured Using Conventional Technologies and 3D Printing—Characterization and Application

One of the breakthrough achievements in surface engineering was the use of nanomaterials for surface modification. The beginnings of nanotechnology date back to the 1950s. Modern nanotechnology began in 1981, when a scanning tunneling microscope was used to observe and manipulate individual atoms. Since then, there has been an exponential growth in research in this field. Currently, nanotechnology is a multidisciplinary science and part of fields such as materials science, mechanics, electronics, biology, and medicine. Nanomaterials are widely used in numerous engineering solutions, facilitating the achievement of the intended functional characteristics of products.

The finishing surface of metal 3D parts is an important part of the technology and even gives proper functional properties to the final products. Ultrasonic Nanocrystal Surface Modification (UNSM) is an innovative surface treatment technique that uses ultrasonic frequency vibration with low amplitude. This action is designed to generate plastic deformation on the surface of 3D-printed metals to improve surface properties and performance [90]. Studies have shown that, following the application of UNSM, the surface roughness (Ra) of AlSi10Mg alloy decreased from 18 to 3.5 μm. The reduction in surface roughness increased corrosion and fatigue resistance. UNSM machining is believed to have great potential in processing 3D-printed metals.

Three-dimensional printing technologies have a wide range of applications including drug delivery systems, scaffolds for tissue engineering, and the fabrication of electronic devices [91]. As technology improves, scalability in the 3D printing field continues to decrease in favor of better surface quality. The micro/nanoscale in 3D printing, especially in the evolution of biomedicine and electronic devices, is playing and will continue to play an increasingly important role, in particular in obtaining hydrophobic surfaces.

**Figure 7:**
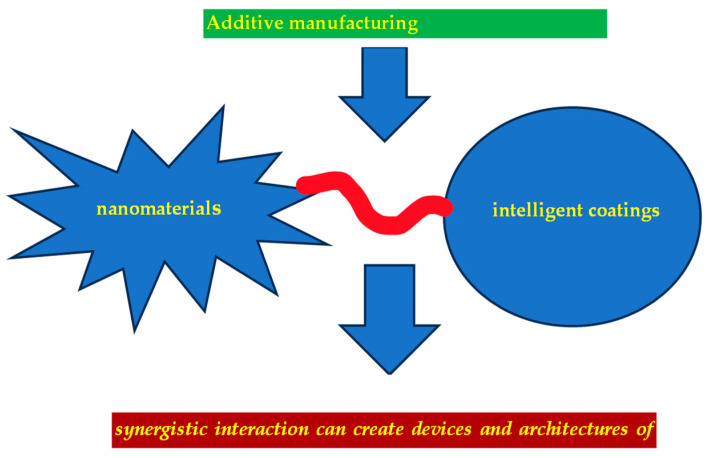
Synergistic interaction 3D printing with nanomaterials and intelligent coating can create unprecedented effects of produced goods.

The manufacture of products by 3D methods from nanomaterials was studied by Elder et al. [92]. They found that the use of nanomaterials in the specific mechanisms of 3D printing technology often limits its potential applications. However, in the final conclusion, the researchers recognize that the limitations can be overcome by developing the ability to model and control nanomaterials in the 3D printing process. The synergistic interaction of these processes enables multiscale additive manufacturing, which can create devices and architectures of unprecedented complexity and broad functionality (Figure 7).

Carbon-based nanomaterials, such as carbon nanotubes (CNTs) and graphene, have unique structural, electrical, and mechanical properties, making them ideal candidates for improving the functionality and durability of traditional metal products and additively manufactured components [93]. Coating metal surfaces with carbon nanomaterials creates new application possibilities. For example, single-walled carbon nanotubes have applications that enhance the cytocompatibility of 3D-printed bone scaffolds made of titanium alloys or biomedical steel.

Three-dimensional printing in addition to conventional technologies is a contemporary method in biomedicine that is being used more and more widely. Three-dimensional printing enables the fabrication of versatile scaffolds with complex shapes to ensure the homogeneous distribution of cells or other compounds trapped in the scaffold, and allows precise control of pore size and shape, porosity, and pore interconnections that contribute to the structural stability of the structure. The use of magnetic nanoparticles in these solutions is currently under investigation due to their unique properties and biocompatibility [92]. Fe_3_O_4_ NPs in combination with hydroxyapatites improve the performance and attachment of cells to structures [94]. In addition to these particles, CuFeSe_2_ nanoparticles have also been attempted to be used in 3D-constructed bone frameworks [95]. Such scaffolds have been found to stimulate new bone formation in bone defects.

Nanotubes are used in 3D printing to produce electrical components, conductive polymer nanocomposites with enhanced mechanical properties [96]. Various metallic nanoparticles (NPs), including gold, silver, copper, and titanium, have been studied for use in 3D printing. Gold, in particular, has been used in biomedicine in implants and dentistry due to its biocompatibility [97]. There are prospects for using nano-gold in the field of cardiac tissue engineering [98]. Also, nano-copper is being explored for use in biomedical solutions produced by additive manufacturing, similar to nanometric titanium [99]. Nanometric iron oxide (Fe_3_O_4_) has shown promise in 3D-manufactured PCL/Fe_3_O cancer-fighting products [100]. The use of metallic nanomaterials in 3D-printed parts requires further in-depth research.

A review paper [101] presents a range of possibilities for the production and application of devices fabricated with 3D technologies from nanometric materials. Three-dimensional technology enables free fabrication of devices based on nanomaterials, providing unique optical, chemical, mechanical, and electrical properties compared to passive designs.

Elder et al. [92] highlighted the synergistic effect of nanomaterials interacting with 3D technology. The researchers concluded that such interaction creates tremendous opportunities to produce materials and devices with multifaceted advantages over conventional solutions. The authors present various fields in which nanometric products made with 3D technology can be used and outline the distinguishing features of such products. The unique properties of nanomaterials and their attractive features as building blocks for programming and modulating a wide range of functions for 3D architecture and devices are not yet completely recognized and exploited at present. The fields where the prospective use of the unique properties of 3D nanoproducts may take place are primarily biomedicine and electronics.

The application of 3D technology and nanomaterials in biomedicine and electronics is also presented in the work of Muldoon et al. [102]. Three-dimensional technology allows for the development of devices at the micro- and nanoscale and creates prospects for further device development in these fields.

Nanomaterials with small dimensions and controlled microstructures are characterized by exceptional antifouling performance. In particular, the use of nanocarbon membranes in this field is promising [103]. In the work by Bouranta et al. [104], using 3D technology, styrene (ABS)/micro-ZnO or nano-ZnO composite meshes with different metal oxide contents were fabricated and their antifouling effect was investigated. The results yielded promising data but the researchers conclude that further improvements to the fabricated products are needed.

**Figure 8:**
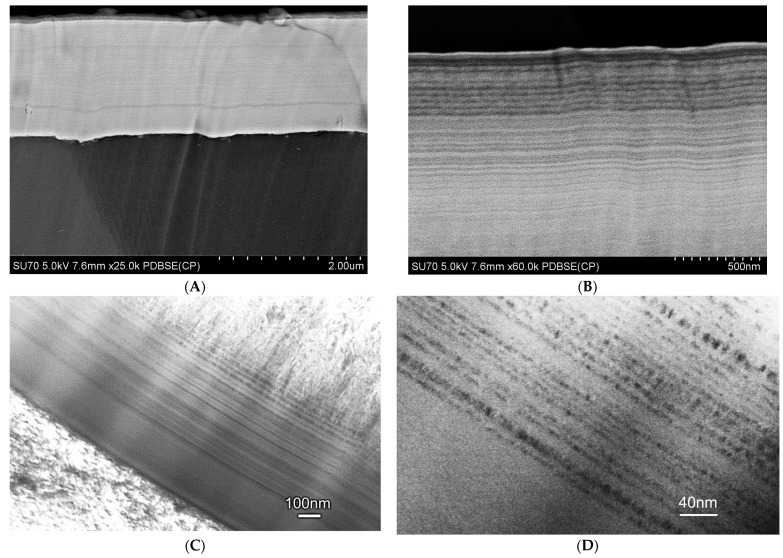
Nanometric layered structure of Ni-Cr_3_C_2_ coating deposited using the ARC PVD method, (**A**,**B**) scanning and (**C**,**D**) transmission electron microscope JEM2010ARP (TEM).

PVD coatings are one of the most frequently used for anti-wear protection of metal surfaces. Hard protective coatings of chromium and tungsten carbide show excellent physical properties such as high hardness, strength, toughness, chemical stability, and corrosion resistance. In the work [105], the possibility of producing nanometric coatings of chromium carbide on an Al-Si aluminum alloy substrate was investigated. The deposition of Cr_3_C_2_ carbide coatings was the essential goal of the performed investigations. It was found that the structure of chromium carbide coatings produced using the Arc PVD method had nanometric features. The coating consists of alternating layers of Ni/Cr-CrxCy with a width of less than 100 nm (Figure 8).

Nanostructured PVD coatings are also used in medicine. In the work of Noori et al., Ti6Al4V titanium alloy implants were covered in a TiN and ZrN single layer and CrN/TiN and CrN/ZrN nanostructured multilayer by means of the ARC PVD technique. All surfaces had satisfactory bioactivity. In all, this study showed that a CrN/ZrN nanostructured multilayer thin film is a potentially suitable candidate for orthopedic and dental implants.

The TiAlCrSiYN-based family of PVD (physical vapor deposition) hard coatings was specially designed for extreme conditions involving the dry ultra-performance machining of hardened tool steels. In the work of Chowdhury et al. [106], different architectures of TiAlCrSiYN-based coatings were investigated: (a) monolayer; (b) multilayer; and (c) bi-multilayer with varying thickness of the multilayer. The abrasion resistance of the produced coatings was tested.

In the work of Góral et al. [107], the erosion resistance of multilayer Cr/CrN coatings deposited using the ARC-PVD method was tested in industrial conditions for use on aircraft engine compressors. A multilayer coating with Cr/CrN microlayers with a thickness of approximately 3 mm was produced. The individual microlayers were approximately 300 nm thick. The tests showed high resistance of the produced coatings to erosion, which creates prospects for using these coatings to protect aircraft components.

Among the anti-wear coatings, the most popular are coatings based on titanium nitride, titanium carbonitride, and titanium aluminum nitride and carbide coatings made of chromium carbides or tungsten carbides [108]. However, diamond and carbon coatings are becoming more and more important, reducing wear, friction, and corrosion, e.g., diamond-like carbon coating, which, compared to conventional coatings, has better anti-adhesive properties and quite high elasticity [109]. The use of an appropriate coating in combination with the tool material can also give it unique features that are not possible to obtain using traditional materials.

Nanometric coatings produced using the PVD method have numerous applications in the metal industry due to the well-mastered coating deposition technology and a significant variety of materials that can be used to cover surfaces [110]. The CVD method, on the other hand, is a more sophisticated technology due to the much higher temperatures required to deposit the coatings than in PVD [111]. Its practical dissemination is much lower than that of PVD technology. Currently, it is used primarily for applying coatings to tools made of sintered carbides and ceramic materials. However, the CVD system also has a wide range of applications in many different areas of the economy. CVD technology is used to deposit coatings for medical applications [112]. Using the CVD method, thin films and complex layered micro- and nanostructures are deposited in semiconductor devices [113]. CVD technology isa low-cost, high-throughput method to make glass coatings [114,115]. Semiconductor materials deposited by CVD are widely used in optoelectronics devices such as light-emitting diodes and infrared detectors [116]. Transparent Conductive Oxides can be coated on soda–lime glass sheets and on a wide variety of metal foils and plastics by CVD [117]. It is commonly used primarily for applying coatings to tools made of sintered carbides and ceramic materials [118]. 

The most common method of depositing coatings is thermal spraying, which has many varieties. It is much less complicated than PVD and CVD. Thermal spraying can be carried out using a special gun or robots, which allows coatings to be deposited on the production floor. Work on coatings produced by thermal spraying focuses on research relating to industrial applications.

**Figure 9:**
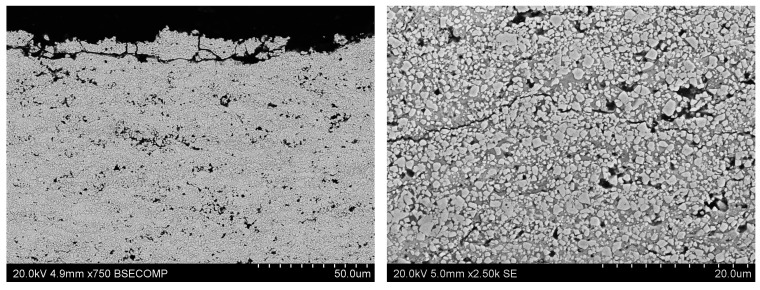
WC-Co-CrC-Ni coating structure prepared by HVOF.

In the work [119], the use of thermally sprayed coatings for anti-friction protection of industrial fan blades is presented. The research concerned coatings made of Cr_3_C_2_-NiCr and WC-Co powders. Additionally, the compositions NiCrSiB + 5%Fe nanoparticles, NiCrSiB + 5%Cr nanoparticles, NiCrSiB + 2.5%Fe + 2.5%Cr nanoparticles were tested. The most advantageous coating for anti-erosion protection turned out to be the WC-Co-CrC-Ni coating produced by the HVOF method (Figure 9).

A review article on the latest developments in thermal spray technologies [120] presents the applications of coatings deposited by thermal spraying in various areas of the economy. The authors pay attention to medical applications such as the deposition of nano hydroxyapatite (HA) coating on commercially pure titanium (Cp-Ti).

Thermal spraying is used to deposit anti-corrosion coatings on equipment used in the nuclear industry. Thermally sprayed coatings are also used in the automotive and aviation industries and in many other industrial sectors [118,121,122,123,124,125].

Galvanic coatings were first used in 1836 to coat steel with zinc. Galvanization is an electrolytic method of producing coatings on various materials. This process involves transferring ions from the coating material to the metal in an electrolytic bath. Galvanic coatings are still very popular, including in the mechanical engineering sector, where they are highly valued for their anti-corrosion properties and the ability to control surface properties. The potential of galvanic coatings is wide and continues to evolve [126]. Research has been carried out to increase the corrosion resistance of coatings protecting devices exposed to changing weather conditions [127]. In the work [128], the research concerned the galvanic corrosion of materials used to produce rifles. It was found that samples coated with a Zn phosphate layer showed the best resistance in all corrosive environments. Galvanic coatings in particular are associated with corrosion phenomena. They are coatings commonly used to protect surfaces from corrosion, which is a leading issue in many aspects of industrial applications [129]. The development prospects of this technology are mainly associated with the improvement of galvanic coatings and their adaptation to current surface protection principles. Galvanic coatings are often found as part of hybrid coatings used by various surface engineering technologies, including coatings in the automotive, aviation, and other metal industries [130,131,132,133,134,135] (Table 2).

## 5. Surface Engineering Market

The degradation of materials due to wear and corrosion is estimated to be very high. Through the possibilities of surface protection, this phenomenon can be prevented to some extent, thus reducing operating costs. The manufacture of coatings, surface refinement, and surface modification, in addition to their protective function, also provide new features and properties for materials and manufactured devices. Nanomaterials in conjunction with 3D technology often make it possible to achieve unique properties not achievable with conventional technologies.

Automotive and motorsports are some of the most advanced fields in the modern world, where the latest technological solutions are being applied, including surface engineering and 3D printing [90].

With advanced manufacturing methods and equipment, it is possible to reduce friction, improve performance, and obtain better maintenance and durability of components and parts in automobiles. In particular, in the world of auto racing, it is extremely important to ensure the quality of the components used. In the case of racing engines, ordinary parts are being replaced with high-end parts. Innovations concern the following:

- Engines (camshaft, crankshaft, valve lifters, valves, pistons, and suspension arms);
- Braking system (disk, pads, and drums)—transmission components (clutch, gears, differential, and pressure plate);
- Steering system.

The automotive surface treatment market refers to electroplating, anodizing, painting, and coating, the purpose of which is to protect automotive surfaces from corrosion, wear, and environmental factors (Table 3). The economy, as well as the growing emphasis on lightweight materials in the automotive industry, is driving increasing demand for esthetically pleasing and corrosion-resistant automotive components. The automotive surface treatment market plays a key role in improving the overall quality and functionality of vehicles, thereby contributing to the durability and performance of automotive components.

Technological advances in surface treatment processes such as plasma treatment, nanocoatings, and electroplating are expected to drive the growth of the automotive surface treatment market. These innovative solutions offer superior performance and cost-effectiveness, leading to their widespread use in the automotive industry [136].

The important role of surface engineering in the automotive industry translates into economic impact. The global automotive surface treatment market is expected to grow at a significant rate during the forecast period, between 2023 and 2030. In 2022, the market is expected to grow at a steady pace. With the increasing adoption of development strategies by key players, the market is expected to rise even beyond the forecast horizon [137].

The automotive surface treatment market size is projected to reach multi-million amounts by 2029, compared to 2022, at an expected CAGR between 2022 and 2029. Based on product types, the market is segmented on the basis of the largest share of the automotive surface treatment market. In 2023, it was anti-rust oil treatment, electroplating, electrophoresis and powder coating, and surface spraying. The leaders in the automotive surface treatment market are North America (United States, Canada, and Mexico), Europe (Germany, United Kingdom, France, Italy, Russia, Turkey, etc.), Asia Pacific (China, Japan, Korea, India, Australia, Indonesia, Thailand, Philippines, Malaysia, and Vietnam), South America (Brazil, Argentina, Colombia, etc.), and the Middle East and Africa (Saudi Arabia, United Arab Emirates, Egypt, Nigeria, and South Africa).

The category of exterior surface materials includes various forms, such as coatings, paints, finishes, and specialty materials. These materials, among others, are applied to the exterior surfaces of vehicles to protect against corrosion, environmental conditions, weathering, abrasion, and other environmental influences. In addition, the application of such materials improves the appearance, strengthening the brand and perception of the vehicle.

According to Zion Market Research, the global market for surface materials used in transportation is expected to experience significant growth. In 2023, the market was valued at USD 32.46 billion and is expected to reach USD 68.27 billion by the end of 2032. This implies a compound annual growth rate (CAGR) of about 10% between 2024 and 2032. For example, the United Kingdom Paints and Coatings Market was valued at USD 2.76 billion in 2023 and is anticipated to project robust growth in the forecast period with a CAGR of 3.84% through 2029 [138].

Electroplating has regained its position as one of the most important technologies in the industry for many years.

The global automotive paints and coatings market size was valued at USD 15.65 million in 2022 and is expected to grow from USD 16.32 million in 2023 to USD 22.86 million by 2031, at a CAGR of 4.3% during the forecast period (2024–2031). Demand for automotive paints and coatings is directly affected by the growth of the global automotive sector.

The automotive paints and coatings market, valued at USD 14.56 billion in 2023, is experiencing rapid growth, projected to exceed USD 17.86 billion by 2032, at a compound annual growth rate (CAGR) of 2.30% over the forecast period. In contrast, the global 3D printing market in 2024 is USD 17.5 trillion and is expected to reach USD 37.4 trillion in 2029 [139].

**Figure 10:**
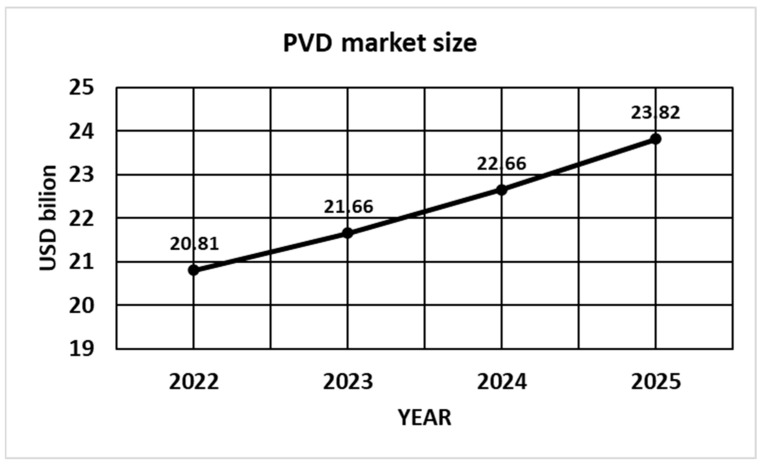
PVD market size, with the forecast.

The growth of the global physical vapor deposition market size is presented in Figure 10. In 2022, its value was USD 20.81 billion, in 2023 it was USD 21.66 billion, in 2024 is it projected to grow to USD 22.66 billion, and in 2025 23.82 billion [140]. It is expected to surpass around USD 38.47 billion by 2032 with a registered CAGR of 6.6% during the forecast period 2023 to 2032. The U.S. physical vapor deposition market size was valued at USD 3.4 billion in 2022.

**Figure 11:**
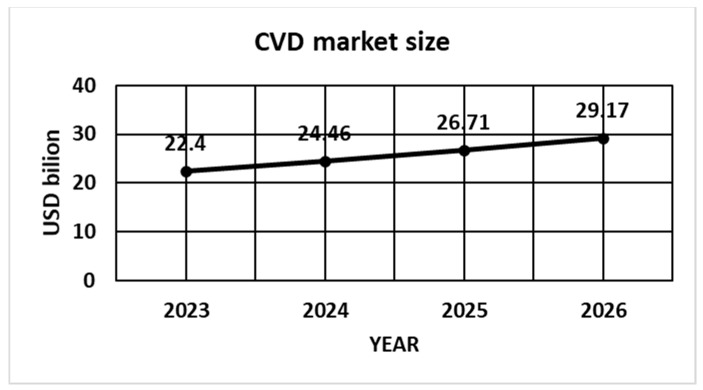
CVD market size, with the forecast.

The global chemical vapor deposition market size accounts for USD 24.46 billion in 2024 and is expected to be worth around USD 58.98 billion by 2034, at a CAGR of 9.2% from 2024 to 2034 [141] (Figure 11).

The global thermal spray coatings market size was valued at USD 10.72 billion in 2021 to USD 11.25 billion in 2022. The global thermal spray market size was valued at USD 11.8 billion in 2023. It is estimated to reach USD 16.36 billion by 2030, growing at a CAGR of 4.8% during the forecast period (2023–2030) [142].

The 3D and 4D Technology Market size is estimated at USD 295.35 billion in 2024, and is expected to reach USD 915.66 billion by 2029, growing at a CAGR of 21.82% during the forecast period (2024–2029) [143]. In 2024, the 3D and 4D Technology Market size is expected to reach USD 295.35 billion. The 3D Systems Corporation, Dolby Laboratories, Inc., LG Electronics Inc., Barco N.V., and Samsung Electronics Co., Ltd. are the major companies operating in the 3D and 4D Technology Market. In 2024, North America accounts for the largest market share in the 3D and 4D Technology Market [144]. Asia Pacific is estimated to grow at the highest CAGR over the forecast period (2024–2029), and is the fastest growing region in 3D and 4D.

Three-dimensional-printed products are made of layers that are not as strong as parts produced using conventional techniques such as injection molding. Additionally, there is a demand for larger product sizes than those that can be produced using 3D printing. Therefore, the ability of this technology to revolutionize mass production is still a long way off. Today, 3D printers are still largely used to create one-off prototypes or small-scale prints. Therefore, the cost, availability, and material issues associated with 3D printing technologies hinder market growth.

Printing with metallic powders is playing an increasingly important role in automotive applications, using a number of specialized technologies that require a strictly defined size and high-quality pellets [145]. Currently, most experts’ interest in AM technology is almost entirely focused on metal printing, and the industry is not considering any (except perhaps ceramics) alternatives for metal applications in AM as structure complexity and cost increase.

Thanks to the availability of advanced 3D printing technologies, affordable costs and increasingly better technical performance, desktop printers are now being used by hobbyists and professionals to develop functional parts, especially consumer products. This market is expected to grow even faster than the 3DP field as a whole. Three-dimensional printing is changing the rules of the game in the market. It allows for faster innovation: it is unimaginably faster compared to the rate of change of using traditional manufacturing methods. For example, the two-year time to bring a product from idea to market can be reduced by 3D methods to two months. This is due to features such as the use of personalization, product optimization, manufacturing efficiency, and the introduction of new business models. Future breakthroughs in 3D printing will occur at the intersection of many fields of science and technology. They should involve a combination of advanced high-performance printing technologies, new 3DP-specific software (DfAM), and new materials for the rapid production of 3D objects that often cannot be made using other methods.

The emergence of 3D technology has become a breakthrough event that has created entirely new production opportunities in numerous areas of the economy [146]. Some limitations of this technology are also reported [147]. These include limitations on the materials used, high production costs, dimensional limitations, stability, precision, and others. Noteworthy are its features such as flexibility, speed, efficiency, and low costs once production is implemented. It is possible to list the areas of the economy in which 3D printing is increasingly used, such as the automotive industry, aviation, medicine, electronics, and others. In some areas, the use of 3D printing is gaining ground. The technology is relatively young and evolving, undergoing continuous improvement. The implementation of 3D printing technology in an organization does not only involve the purchase of appropriate equipment; it is necessary to ensure the appropriate level of knowledge [148]. Undoubtedly, 3D printing plays an extremely spectacular role in the health sector [149]. The integration of 3D printing with conventional technologies appears to be the most forward-looking [150,151,152,153,154,155,156,157]. In particular, this refers to technological developments in Industry 4.0 [158,159] and Industry 5.0 [160,161,162]. Advanced users are investing in 3D printing technology and expect increased reliability and performance from their printers. The advantages of additive manufacturing are being increasingly recognized and more widely applied on a larger industrial scale. With the development of this technology, it is also expected that there will be a continued increase in demand for training and education in this industry, both face to face and online [163].

In the UK, where 3D technology is one of the most strongly developed, several teams have been formed to work on analyzing the factors limiting the development of 3DP/AM. Important areas currently being developed include, in addition to the introduction of new processes and materials, research and standardization of the materials used for 3D printing, the products and processes used, as well as safety conditions. The latter particularly concerns the aerospace industry and medical applications.

## 6. Summary

One of the first assessments of products is their visual quality. For this reason, the final surface treatment is of great functional and decorative importance. This applies in particular to metal products. Most everyday objects are made of metals; they dominate in the automotive industry, aviation, railways, and are present in almost every area of life and the economy. For the needs of the metal industry, a dynamic field of surface engineering has been developed, using the latest scientific achievements in the production of various types of coatings. Both classic surface treatment and modern methods of surface refinement will improve the functionality and quality of products. The development of hybrid coatings, intelligent coatings, and nanometric coatings is the result of numerous studies by entire scientific teams and the subsequent implementation of the obtained results into industrial technologies. The expected development of the industry towards Industry 4.0, 5.0, and even 6.0 will show an increasing demand for innovative, intelligent solutions which, regardless of the market situation, must be based on new scientific achievements in the field of materials engineering. Synergistic interactions of surface engineering technologies combined with other areas of science and the economy have a chance of achieving the goals set in Industry 5.0 (Figure 7). Metal technologies in the automotive industry require the use of hybrid coatings. Galvanic anti-corrosion coatings are of great importance, being part of a set of layers creating coatings, whose task is to protect the metal against the oxidizing effects of the environment (Table 2).

Anti-wear coatings used on devices, elements, and tools constitute a hugely expanded branch of the surface engineering market, extending the life of tools (Table 1, Figure 8). In the aviation industry, an undoubted achievement is superhydrophobic coatings that protect the surface against icing, which can also be used for ship bodies. Coating glass surfaces with superhydrophobic surfaces enables the use of self-cleaning windows. Glasses and their surface treatments create numerous new application possibilities. Electronics benefits greatly from the ability to deposit thin nanometer layers on semiconductors and other electronic components. Great progress has been made in biomedicine. Implants, whether made using traditional technologies or innovative technologies, require surface treatment. Hydrophobic surfaces are of great importance in medicine because they create antibacterial coatings. Thanks to their properties, they ensure cleanliness and insulation and make it difficult for dirt and bacteria to adhere to the surface. Surfaces covered with such coatings are easier to keep clean, which may help reduce the risk of spreading bacteria and viruses. Nanocoatings appeared as a result of new material discoveries. Changing the size of the structure to less than 10 nm changes the properties of the material. Nanometric metallic particles in anti-erosion coatings increase hardness and wear resistance. Nanocarbon coatings change surface properties, electrical conductivity and other functional properties. The association of nanotechnology with 3D printing has resulted in the development of coatings used on the surfaces of elements produced by 3D printing. Thanks to the synergy of the impact of nanotechnology and 3D printing, products gain new functional features. The surface of products produced using the 3D method becomes hydrophobic and superhydrophobic, and is also strengthened and smoothed. Due to the porosity of 3D surfaces, in many cases the surface treatment is of decisive application importance. This is particularly important in medicine, where 3D technology has taken a prominent place. For example, today implants and other components used in dentistry are mostly made with 3D printing. All crowns and artificial teeth can be made using this technique. The use of 3D printing enables accurate reproduction of the printed dental element, significantly shortens its production time, and creates structures from any material. Three-dimensional printing also replaces the classic production of medical implants. It enables the production of implants from any material with very accurate shape reproduction. Due to such revolutionary changes in implantology, technologies for developing their surfaces have been developed so that 3D products meet medical requirements by depositing layers and coatings. The surface technology market is constantly growing and expanding with new technological solutions. The Global Protective Coatings Market size is expected to grow from USD 5.4 trillion in 2022 to USD 7.0 trillion by 2027, at a GAGR of 5.5% during the forecast period. This indicates a broad and constantly growing market demand for surface engineering technology products. Recent achievements related to the development and production of intelligent coatings demonstrate the possibility of further development of this industry. Surface engineering has become an integral part of the production of products manufactured in almost every field of the economy. It plays an important role in assessing the quality of metal products, especially including those manufactured with 3D technology.

The most important elements of current surface engineering and the expected directions of development in this field include the following:

1. Skillful use of the latest technical and scientific achievements in coating production techniques.
2. Automation of coating application processes.
3. Based on knowledge from many fields of science, in particular on processes taking place at the atomic level.
4. A high degree of usability and practical use, increasing the functionality of coated products.
5. Putting a lot of effort into making surface technologies as environmentally friendly as possible.
6. In the future, the development of surface coating technology will probably focus on the needs of the metal industry, which is key to consumer production.
7. The development of surface technologies will be an integral part of Industry 5.0, the premise of which is to act for the benefit of consumers.
8. Industry 5.0 reduces waste, which is reflected in technologies such as 3D printing. By using cheaper recycled metals and then covering them with functional coatings, the products can be used in various environmental conditions.

## Figures and Tables

**Table 1:** Coating deposition methods—PVD, CVD, TSC, and galvanic coating—sample structures, and advantages and disadvantages of methods.

Psyhical Vapor Deposition (PVD)	PVD is characterized by a process in which the material goes from a condensed phase to a vapor phase and then back to a thin film condensed phase. Advantages—PVD coatings are thin layers and range from 1 to 15 microns, it is the most common technology for treating metal surfaces, precise control of coating thickness, very good adhesion of the coating to the substrate, high smoothness of the coating, resistance to damage, scratches, discoloration, and abrasion, extremely hard and scratch-resistant, a large selection of surface coating materials, thermal shock resistance. Disadvantages—the complexity of coating equipment, high process requirements and long coating times, susceptibility to internal stresses and microcracks due to different shrinkage of the coating and the substrate during cooling.	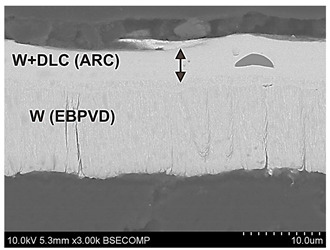 Tungsten coating deposited by electron beam physical vapor deposition (EBPVD) method, on the top W+DLC coating deposited by Cathodic Arc Deposition—(ARC PVD) method
Chemical Vapor Deposition (CVD)	CVD is a materials processing technology in which thin films are formed on a heated substrate via a chemical reaction of gas-phase precursors. Advantages—great resistance to high temperatures, high mechanical strength, very good adhesion to the substrate. Disadvantages—the surface is not as smooth as PVD, so the workpiece may stick, susceptibility to thermal shock compared to PVD coatings, the thickness of the coating causes a large rounding of the cutting edge, although this is not always a problem, the complexity of coating equipment, the process is often used in the semiconductor industry to produce thin films, high process temperature reduces the impact strength of the tool in the surface area.	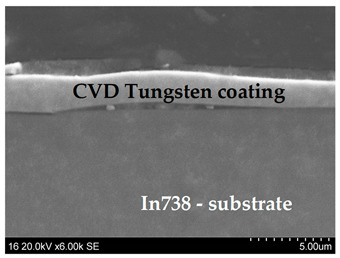 Tungsten coating on In 738 substrate deposited by CVD method
Galvanic Coatings (GCs)	Galvanic coatings are created in the galvanization process (or more precisely, electroplating), which involves creating coatings using an electrolytic method, most often on metal products. Advantages—coatings can be made of most metals and alloys, high durability of galvanic coatings, high resistance to corrosion and material abrasion, possibility of applying the technology to elements with extremely complex shapes, improving the thermal or electrical properties of processed parts, excellent visual effect for decorative coatings, electroplating treatment is widely used in industry. Disadvantages—the galvanization process requires prior preparation of the treated surface, which most often involves degreasing and cleaning it, the need to use chemicals that are often harmful to the environment, defects may appear, e.g., those resembling orange peel, if the process is not carried out properly.	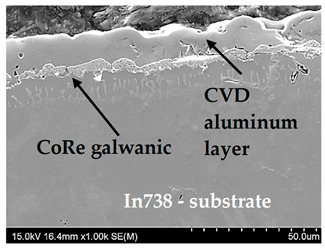 Galvanic coating on an In 738 substrate—CoRe galvanic coating (3 µm) and et the top CVD aluminum layer
Thermal Spray Coating (TSC)	In TSC technology, the coating material in the form of wire or powder is delivered to the metallization device, where it is melted and then directed at a high speed in a plasma stream to the substrate to form the coating. Advantages—widely used, the technology enables the spraying of thermally sensitive materials and very different material combinations because the adhesion mechanism is purely mechanical, high thermal and electrical conductivity of coatings, high density and hardness of coatings, high uniformity of coatings, no melting, possibility of spraying fine particles (5–10 μm), possibility of spraying nanomaterials and amorphous materials, minimal surface preparation, low energy consumption, possibility of obtaining complex shapes and internal surfaces, high efficiency thanks to high power factor, high settling rates and efficiency, no toxic waste, increased operational safety due to the absence of high-temperature gas streams and radiation. Disadvantages—almost zero plasticity in the sprayed state, the need for a plastic substrate, difficulties in processing pure ceramics and some alloys such as hardening alloys, high cost of helium, nozzle contamination and erosion, should not be used in conditions of high point loads (e.g., ball and needle bearing races) and impacts, coatings may be porous.	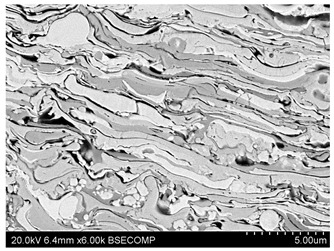 WC-CrC-Ni coating deposited by thermal spraying method

**Table 2:** Hybrid coating on car body.

Base Coat (Paint Color)	~10–25 Mikrons, Vehicle Color Layer
Primer	~20–30 mikrons creates uniform and smooth surface for base coat, promotes layer adhesion
E-COAT	~20 mikrons, deposited only on metal, corrosion protection
Phosphate	~5 mikron, deposited only on metal, corrosion protection, promotes adhesion
Substrate	(metal, plastic, carbon fiber, etc.)

**Table 3:** Surface protection systems in the automotive industry.

anodizing	various colors but predominantly silver or black is used for the automotive trade; with the increased use of aluminum, anodizing has also significantly increased in automotive applications
powder coating	powder coating offers many advantages over the traditional painting process: blocks and heads can be powder-coated immediately after the casting process, which avoids the cleaning step, the powder adheres to raw castings without the need for pre-treatment, and the resins used in powder coating have a higher molecular weight, which ensures a higher quality finish of machined parts.
chrome-free chemical conversion coating	car conversion coatings consist of layers of materials that are chemically applied to vehicle body structures prior to painting to improve corrosion protection and increase paint adhesion; these coatings are a consequence of chemical reactions on the surface and are placed between the paint layers and the base metal
E-coating (electrical immersive painting)	an electrocoat system applies a DC charge to a metal part immersed in a bath of oppositely charged paint particles; the paint particles are drawn to the metal part and paint is deposited on its surface, forming an even, continuous film over every curve, crevice, and corner, until the electrocoat reaches the desired thickness.

## Data Availability

Not applicable.
